# A systematic scoping review of group reflection in medical education

**DOI:** 10.1186/s12909-024-05203-w

**Published:** 2024-04-10

**Authors:** Gillian Li Gek Phua, Jasmine Lerk Juan Owyong, Ian Tze Yong Leong, Suzanne Goh, Nagavalli Somasundaram, Eileen Yi Ling Poon, Anupama Roy Chowdhury, Simon Yew Kuang Ong, Crystal Lim, Vengadasalam Murugam, Eng Koon Ong, Stephen Mason, Ruaridh Hill, Lalit Kumar Radha Krishna

**Affiliations:** 1 Yong Loo Lin School of Medicine, National University of Singapore, NUHS Tower Block, Level 11, Singapore, Singapore; 2 Division of Supportive and Palliative Care, National Cancer Centre Singapore, Singapore, Singapore; 3 Lien Centre for Palliative Care, Duke-NUS Medical School, National University of Singapore, Singapore, Singapore; 4 Division of Cancer Education, National Cancer Centre Singapore, Singapore, Singapore; 5 Duke-NUS Medical School, 8 College Road, Singapore, 169857 Singapore; 6 School of Humanities and Behavioural Sciences, Singapore University of Social Sciences, 463 Clementi Road, Singapore, Singapore; 7 KK Women’s and Children Hospital, 100 Bukit Timah Rd, Singapore, 169854 Singapore; 8 Division of Medical Oncology, National Cancer Centre Singapore, Singapore, Singapore; 9 Division of Geriatric Medicine, Singapore General Hospital, Singapore, Singapore; 10 Medical Social Services, Singapore General Hospital, 16 College Road, Block 3 Level 1, Singapore, 169854 Singapore; 11 Assisi Hospice, 832 Thomson Rd, Singapore, Singapore; 12 Office of Medical Humanities, SingHealth Medicine Academic Clinical Programme, 31 Third Hospital Ave, Singapore, 168753 Singapore; 13 Palliative Care Institute Liverpool, Academic Palliative & End of Life Care Centre, Cancer Research Centre, University of Liverpool, 200 London Rd, Liverpool, L3 9TA UK; 14 Health Data Science, University of Liverpool, Whelan Building, The Quadrangle, Brownlow Hill, Liverpool, UK; 15 PalC, The Palliative Care Centre for Excellence in Research and Education, PalC c/o Dover Park Hospice, Singapore, Singapore; 16 Centre for Biomedical Ethics, National University of Singapore, Singapore, Singapore

**Keywords:** Reflection, Group reflections, Medical education, Professional identity formation, Undergraduate medical education, Postgraduate medical education

## Abstract

**Background:**

Reviewing experiences and recognizing the impact of personal and professional views and emotions upon conduct shapes a physician’s professional and personal development, molding their professional identity formation (PIF). Poor appreciation on the role of reflection, shortages in trained tutors and inadequate ‘protected time’ for reflections in packed medical curricula has hindered its integration into medical education. Group reflection could be a viable alternative to individual reflections; however, this nascent practice requires further study.

**Methods:**

A Systematic Evidence Based Approach guided Systematic Scoping Review (SSR in SEBA) was adopted to guide and structure a review of group reflections in medical education. Independent searches of articles published between 1st January 2000 and 30th June 2022 in bibliographic and grey literature databases were carried out. Included articles were analysed separately using thematic and content analysis, and combined into categories and themes. The themes/categories created were compared with the tabulated summaries of included articles to create domains that framed the synthesis of the discussion.

**Results:**

1141 abstracts were reviewed, 193 full-text articles were appraised and 66 articles were included and the domains identified were theories; indications; types; structure; and benefits and challenges of group reflections.

**Conclusions:**

Scaffolded by current approaches to individual reflections and theories and inculcated with nuanced adaptations from other medical practices, this SSR in SEBA suggests that structured group reflections may fill current gaps in training. However, design and assessment of the evidence-based structuring of group reflections proposed here must be the focus of future study.

## Introduction

Reflection allows physicians the opportunity to reflect on their actions, recognize how their thoughts, feelings and emotions affect decision-making processes, clinical reasoning, and professionalism [1–6], from which these insights are then integrated into the evolving values, beliefs, and principles (henceforth belief system) that shape the professional development of physicians [7–11]. The critical role reflection plays in the professional identity formation (henceforth PIF) of physicians [12–17] merits further investigation into its different applications in medical education.

One such example of its application, are group or collaborative reflections, which sees reflections shared amongst two or more participants moderated by a facilitator or supervisor, infused with personal and cultural ‘frames of reference’, emotional insights and personal interpretations and consolidated by shared meaning-making [18–26]. The social nature and interaction in clinical education increase the need to improve upon current reflective practices in medical education [27], which are often challenged by the lack of protective time, limited access to trained support in the packed curriculum of healthcare professionals [19, 23–25, 28–30].

Therefore, a review is proposed to provide a consistent understanding on practices in group reflections, and effective guidelines on the design, structuring, assessment and oversight of group reflective practice in medical education. This review aims to answer the questions of “*What is known about group reflections in medical education*?” and “*How are group reflections structured, assessed and supported in medical education*?”.

## Methods

**Fig. 1:**
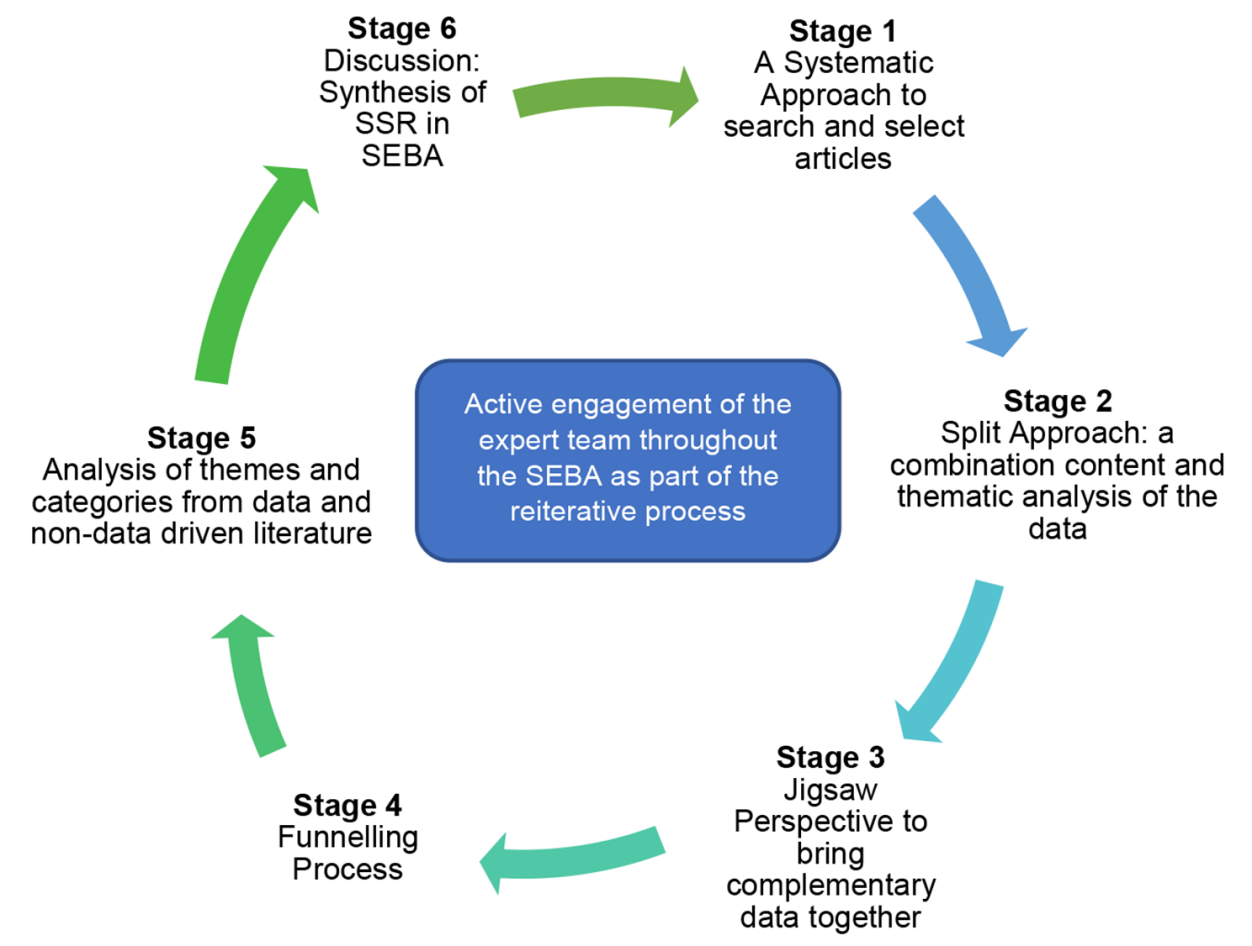
The SEBA process

Krishna’s Systematic Evidence-Based Approach (SEBA) was adopted to guide this systematic scoping review (SSR) (henceforth SSR in SEBA) [31, 32] to identify available data, key characteristics, and knowledge gaps in current concepts of group reflections in regnant medical education literature (Fig. 1). The SSR in SEBA’s constructivist approach [33–40] and relativist lens [41–45] acknowledges reflective practice as a sociocultural construct influenced by the physician or medical student’s narratives, contextual considerations, clinical insights and the program culture and environment.

### Stage 1 of SEBA: systematic approach

#### Identifying the research question

The primary and secondary research questions were determined to be “*What is known about group reflections in medical education*?”, and “*How are group reflections structured, assessed and supported in medical education*?”. These questions were designed around the PICOs (Population, Intervention, Comparison, Outcome, study design) (Table 1).

**Table 1:** PICOs, inclusion criteria and exclusion criteria applied to database search

Group reflections in medical education		
	Inclusion criteria	Exclusion criteria
Population	Junior doctors, residents, specialists, and/or doctors, and/or physicians within the clinical, medical, research and/or academic settings Undergraduate and postgraduate medical students Allied health specialties such as Pharmacy, Dietetics, Chiropractic, Midwifery, Podiatry, Speech Therapy, Occupational and Physiotherapy, Physician Assistants	Non-medical specialties such as Clinical and Translational Science, Alternative and Traditional Medicine, Veterinary Medicine, Dentistry
Intervention	Papers that addressed the incorporation of group reflections for junior doctors, residents, specialists, and/or doctors, and/or physicians, and/or medical students within the clinical, medical, research and/or academic settings Papers that addressed assessment of group reflections	Papers with little detail of implementation or assessment of group reflections in curriculum Papers that evaluated group reflections for purposes other than improving reflective capacity of users
Comparison Outcome	Papers that addressed the following comparisons were also included: Comparison of the various uses of group reflections in different teaching settings Evaluation of the effectiveness of reflections in comparison to other educational interventions Papers that discussed group reflections without the above comparisons were also included. Papers that measured the following outcomes were also included: Impact of the use of group reflections on junior doctors, residents, specialists, and/or doctors, and/or physicians, and/or medical students within the clinical, medical, research and/or academic settings Impact of the use of group reflections on teaching Impact of the use of group reflections on assessment Gaps and improvements to current group reflections programmes	
Study design	All study designs including: mixed methods research, meta-analyses, systematic reviews, randomized controlled trials, cohort studies, case-control studies, cross-sectional studies, descriptive papers, grey literature, opinions, letters, commentaries and editorials Articles in English or translated to English Year of Publication: 2000–2022	Non-English language articles

#### Searching

Members of the research team carried out independent searches from bibliographic databases such as Pubmed, EMbase, Psychinfo, CINAHL, ERIC, ASSIA, Scopus, and Google Scholar, as well as grey literature databases Open Grey, GreyLit, and ProQuest using variations of the term “group reflections”, “group debrief”, and “Collaborative reflections” (Table 2).

**Table 2:** Search strategy for bibliographic databases

Database	Search Strategy
Pubmed, EMbase, Psychinfo, CINAHL, ERIC, ASSIA, Scopus	(“group reflections” OR “group debrief” OR “collaborative reflections”) AND (medical education OR healthcare OR medical training OR clinical practice OR healthcare professionals OR healthcare students)

#### Extracting and charting

Titles and abstracts were independently reviewed by the research team to identify relevant articles that met the inclusion criteria. Full-text articles were independently reviewed, with each reviewer producing their own final list of included articles. Sandelowski and Barroso [46]’s approach to ‘negotiated consensual validation’ was used to achieve consensus on the final list of articles to be included.

### Stage 2 of SEBA: split approach

The ‘Split Approach’ [34, 46–50] was employed to enhance the reliability of the data analyses, which saw the research team split into three groups to independently analyse the included articles.

The first team summarised and tabulated the included full-text articles in keeping with recommendations drawn from Wong et al. [51]’s RAMESES publication standards: meta-narrative reviews and Popay et al. [41]’s “Guidance on the conduct of narrative synthesis in systematic reviews”. Concurrently, the second team analysed the included articles using Braun and Clarke [52]’s approach to thematic analysis whilst the third team of researchers drew categories from Lim et al. [5]’s review entitled “*A systematic scoping review of reflective writing in medical education*” in their employ of Hsieh and Shannon [53]’s approach to directed content analysis.

### Stage 3 of SEBA: jigsaw perspective

Overlaps between categories and themes allowed their combination to create a bigger piece of the puzzle referred to as themes/categories.

### Stage 4 of SEBA: funnelling process

Through the Funnelling Process, themes/categories were compared with the tabulated summaries to determine the consistency of the domains created, forming the basis of the discussion.

### Stage 5: analysis of evidence-based and non-data driven literature

The themes from data-driven or research-based peer-reviewed data were compared to those drawn from grey literature and found to be the same and thus unlikely to have influenced the analysis.

### Stage 6: synthesis of scoping review in SEBA

The Best Evidence Medical Education (BEME) Collaboration Guide and the Structured approach to the Reporting In healthcare education of Evidence Synthesis (STORIES) were used to guide the discussion.

## Results

**Fig. 2:**
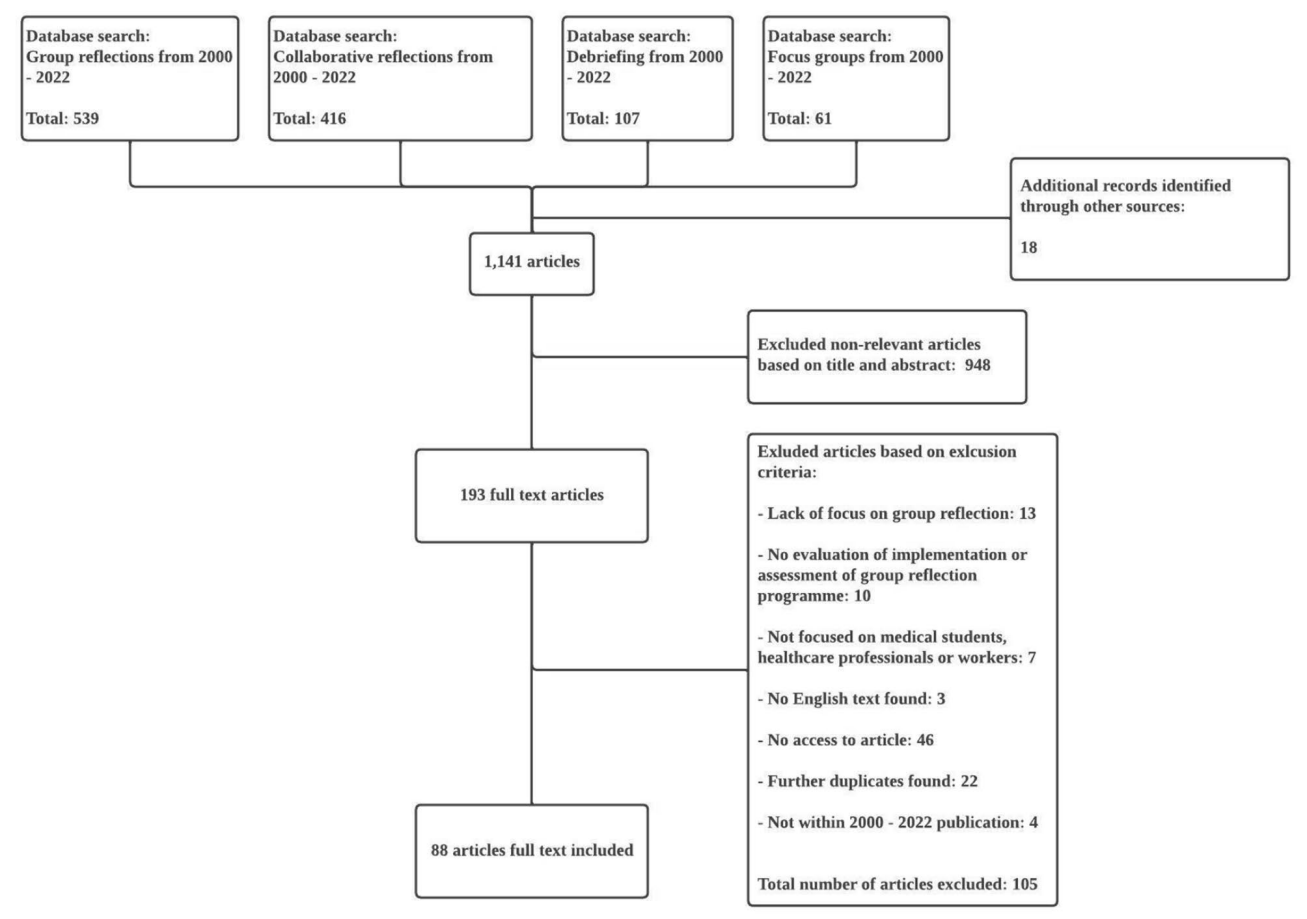
PRISMA flowchart

A total of 1141 abstracts were reviewed, 193 full-text articles were appraised, and 66 articles were included and analysed. The PRISMA flow diagram may be found in Fig. 2.

The participant population and the country of origin are shown in Table 3.

**Table 3:** Demographical data

Year	2000–2005, *n* = 2 [54, 55] 2006–2010, *n* = 7 [25, 28, 56–60] 2011–2015, *n* = 20 [61–80] 2016–2020, *n* = 24 [18, 81–103] 2021-present, *n* = 13 [19–21, 104–113)
Medical students	*N* = 19 [18, 21, 28, 54, 55, 65, 70, 75, 76, 80, 83, 86, 90, 95, 101, 103, 106, 111, 112]
Doctors	• Physicians mentioned, *n* = 18 [25, 61, 63, 64, 73, 81–83, 85, 87, 89, 92, 94, 100, 105, 107, 109, 110, 113] • Residents, *n* = 10 [19, 20, 64, 67, 84, 88, 89, 91, 92, 109]
Others (allied health, nursing etc.)	• Nursing, *n* = 18 [56–59, 69, 71, 72, 74, 77–79, 81, 82, 87, 89, 94, 96, 98, 102, 107–110] • Physiotherapy, *n* = 5 [56, 57, 71, 74, 102] • Psychiatrists, psychologists, *n* = 3 [82, 93, 94] • Pharmacy = 1 [109] • Occupational therapy, *n* = 5 [60, 66, 71, 74, 99] • Midwifery, *n* = 1 [97]
Specialities	• Emergency medicine, *n* = 3 [30, 37, 60] • Paediatric, *n* = 3 [21, 89, 110] • Family medicine, *n* = 2 [64, 113] • Internal medicine, *n* = 4 [67, 76, 91, 92] • Psychiatric, *n* = 3 [68, 93, 94] • Palliative care, *n* = 2 [59, 79] • Obstetrics and gynaecology, *n* = 2 [78, 111] • Radiology, *n* = 1 (100)
Country	United States of America, *n* = 20 [4, 8, 10, 13, 16, 19, 22, 24, 25, 27, 30, 36, 39, 41, 51, 52, 57, 58, 60, 65] Canada, *n* = 6 [60, 66, 74, 83, 84, 113] Sweden, *n* = [69] Netherlands, *n* = 5 [19, 20, 85, 88, 105] United Kingdom, *n* = 9 [18, 57, 65, 79, 80, 87, 100, 110, 111] Norway = 4 [77, 82, 96, 108] Denmark, *n* = 2 [63, 93] Italy, *n* = 1 [25] Ireland, *n* = 4 [58, 72, 97, 98] Finland, *n* = 1 [103] Australia, *n* = 4 [28, 56, 68, 90] Hong Kong, *n* = 1 [55] Iran, *n* = 1 [95] Israel, *n* = 1 [99]

The domains identified were (1) Theories and models, (2) Indications for group reflections, (3) Types of group reflections, (4) Structure of group reflections programs, and (5) Benefits and challenges. Here we consider the data in their entirety and include nurses, allied health professionals, medical students and physicians under the umbrella term ‘clinician’.

### Domain 1: theories and models

Current accounts of group reflections are framed by the traditional concepts and theories employed in individual reflections and reflective writing. These concepts and theories focus on the critique and group discussion of a specific experience and the lessons drawn from this process. However, current concepts also recognise the influence of the clinician’s narratives, clinical insights, belief systems, contextual and environmental considerations as well as their willingness and readiness to share their insights and emotions on the impact on their thinking and practice (Table 4).

**Table 4:** Theories and models of group reflections

Author	Concept
**Reflective Theories**	
Kolb’s experiential learning cycle [104, 114]	It involves a concrete experience, followed by reflective observation and reflection on the experience. It is theorized that abstract concepts and generalizations are formed, which are experimented in future situations, resulting in subsequent new concrete experiences.
Reflexivity [88]	Reflexivity is described as a level of consciousness of ‘cultural, political, social, linguistic and ideologic’ origins of one’s own and others’ voice and perspective. It increases awareness of how personal values and beliefs interconnect with social and environmental contexts.
Mezirow’s transformative learning theory [18]	Encouragement of challenging personal and cultural ‘frames of reference’. In professional dilemmas, one is encouraged to consider alternative responses, focusing on the problem rather than emotions.
Common phases of debriefing [104]	1. **Reaction/ Description** a. Time for participants to diffuse emotions and decompress b. Open-ended questions about participants’ feelings c. Reviewing facts of event 2. **Understanding/Analysis** a. Preview topics/learning objectives b. Explore, discuss inquire c. “What happened and why did it happen?” 3. **Application/Summary** a. Applying learning experiences to a future encounter b. Allow participants to ask questions
Rudolph et al. [104]	• Reaction ⚬ “How did that feel?” to hear initial reactions and validate emotional responses. • Analysis ⚬ Focus on understanding what, why and how actions evolved during the scenario ⚬ Investigates gaps noted during the scenario ⚬ Understand the rationale behind actions ⚬ Work toward closing the gap and reflective discussion ⚬ Use of advocacy inquiry • Summary ⚬ Focus on learning points from the analysis phase and “take away” points
PEARLS debriefing framework [104, 115]	The PEARLS framework is similar to the one outline by Rudolph et al. However, an additional description follows the reactions phase to invite participants to summarize their perspective of the experience to ensure all members and the facilitator are on the same page.
Korthagen’s ALACT model [116]	(1) Looking back on an action (2) Awareness of essential aspects (3) Creating alternative methods of action
“Plus-delta” model [81, 104]	Commonly used as a debriefing model, it involves discussing the positive points from the reflective experience (“plus”) and points that can be improved on (“delta”).

Many theories follow the process of allowing participants to share their reactions to the experience, followed by a deconstruction of the experience through the process of inquiry and discussion [104]. While Kolb’s Learning Cycle served as a baseline for many models for reflection [104, 114], some studies use a combination of models.

### Domain 2: indications for group reflections

Current indications for the employ of group reflections centre on enhancing holistic and collaborative learning [18, 54]. Other indications for group reflections include as a means of determining the nature of the ‘takeaway’ from a specific learning interaction and boosting engagement [19, 20, 99, 117] (Table 5).

**Table 5:** Indications of group reflections

Indication	
Studying change [54] Accessing the hidden curriculum [18, 54] Synthesis of different perspectives [61]. Provide an avenue for formative assessment [105] To increase the social belonging of students [106, 116, 118] Development of a cohesive learning community or social network of support [28, 84, 114, 115, 119, 120] Follow-up to challenging scenarios [81] Provide a learning experience to work through team dynamic issues [86]	• Provide a space and time to examine processes of learning within clinical practice • Draws attention to more subtle, yet important changes in clinical practices. • Illuminates the way clinical decision-making is influenced by knowledge domains, ideas and values underpinning practice. • Provides insights into aspects not amenable using more conventional quantitative methods. • Includes personal and group feedback during the reflective process. • Identifies the motivations behind learning • Identifies, makes sense of and addresses cognitive dissonance during emotionally challenging situations. • Highlight different ways of knowing and learning • Reflective peers can be used as instructional resources as learning intentions and criteria for success are shared. • Group conversations evolve into quasi and reflective thinking, where participants integrate other perspectives into their own. • Stimulates engagement with others which can improve academic performance, health and well-being of students. • A deeper understanding of relevant concepts is co-constructed and co-developing strategies for effective implementation in context. • Deepening and refinement of meaning that the individual and group apply to their practice. • Learning is enhanced through interactive learning and support that occurs in these networks. • As a medium to educate others • To provide emotional relief to those involved with the challenging event. • Particularly useful introduction to dealing with future team conflicts in practice.

Group reflections served a valuable means of accessing the hidden curriculum through facilitating discussions and self-reflection, providing insight into unspoken norms and values which influence clinical reasoning [54]. The synthesis of different perspectives in group reflections also encouraged participants to integrate these diverse viewpoints into their individual understandings of medical practice [61]. As a community of practice, group reflections played an important role in increasing the social belonging of participants through the safe space provided for open dialogue and sharing experiences [106, 116, 118]. This contributes significantly into the development of cohesive learning communities through the co-construction of a shared understanding of relevant concepts and strategies in the clinical context [28, 84, 114, 115, 119, 120].

### Domain 3: types of group reflections

Three distinct methods of group reflections emerged from the data: dialogues, debriefings and focus groups (Table 6).

**Table 6:** Types of group reflections

Type of Reflection	Definition
Dialogues [23–25]	Dialogues are a form of experiential and affective approach to promote new ways of understanding oneself and the world, new possibilities and new questions. They focus on the subjective aspects and encourage the sharing of authority, expertise and perspectives between traditional teachers and learners. These promote reflection and reflexivity by creating space for learners to see one another as equal relational partners, and to question assumptions, power dynamics and structural inequities beyond medicine.
Debriefings [62, 87, 89, 104, 109, 115, 121–125]	A facilitated discussion between 2 or more individuals, revolving around sharing and examining information after a specific event has taken place. Built based on experiential learning theory and reflective practice, it is used to reflect on action and identify areas for improvement. The typical agreed upon process: 1. Emotional reaction. To allow participants to ‘cool down’ and vent strong feelings that may otherwise interfere with the discussion. 2. Analysis. To find out what happened and why. 3. Generalisation. To integrate the simulation experience into real world clinical practice for performance improvement. It is recommended that debriefing takes place immediately after an event, as the immediate recall and availability of those involved will benefit the reflection. • Warm debriefs happen with a slight delay, but within hours of the event occurring. • Cold debriefs occur days or weeks after the event has occurred.
Focus groups [26, 28–30, 120]	A form of group interview aimed at capturing the perspectives of participants in order to explore and generate data on a narrowly focused topic. It usually takes place in a ‘permissive, non-threatening environment’. It can be used at the preliminary or exploratory stages of program development.

Dialogues promote new ways of understanding one’s self and their surroundings, focusing on subjective aspects and facilitating the sharing of perspectives between participants [23–25]. Debriefings are structured discussions which occur following specific events within medical education, serving as a method for reflection on action and identifying areas for improvement [62, 87, 89, 104, 109, 115, 121–125]. Focus groups are utilized for exploring and generating data on niche topics, providing a platform for participants to share insights and contribute to a deeper understanding of experiences [26, 28–30, 120].

### Domain 4: structure of group reflections programs

A range of structures influencing the effectiveness of group reflective programs were uncovered, which encompassed various aspects such as the group size, frequency of meetings, modalities and assessment methods (Table 7).

**Table 7:** Structure of group reflections

Dimensions in structuring group programme	Elaboration/ Examples
Structured vs. unstructured	Structured Preparation • Pre-reading [68] • Introduction to the process [56, 68] Communication of objectives [105] Ground rules • Emphasising confidentiality [104, 105] • Encouraging active participation [104] Providing an explicit invitation to share perspectives [105]. Participants possess some degree of autonomy over reflective content [101, 102, 105] however common reflective themes include • Day to day activities and perspectives [18, 126] • Challenges [127, 128] • Actual case studies [129] • Views and experiences on a program [119] • Suggestions for improvement [21] • Student-patient interaction [68] • Professionalism in practice [61] • Ethical dilemmas [99] • Psychological supervision and clinical reasoning for practice [18].
Size of group	Less than 7 [62, 126, 129–131] More than 7 [21, 119, 132]
Frequency	Once-off [130] Weekly [18] Bi-weekly [132] Every few months [129, 133] 1 h 15 min [126] 90 min [132] 2 h 15 min [18]
Modality of reflections	In person [21, 61, 126, 133] Online [25, 128, 134] Video review [104, 106] Vignettes [61] Case studies [99] TV shows [99] Collaborative writing [99] Word clouds [102] Free discussion [118] Role-play [118]
Activities	Role-playing [128, 134] Vignettes [61] Collaborative writing [135] Clinical observation activity [86]
Assessment	Wellness scales to determine if well-being has improved [106] Questionnaires [25, 130, 132] Feedback survey [18, 86, 101, 105, 109, 116, 133, 134] Informal feedback [62] Written portfolio [18] Thematic coding of discussion transcripts [61] General approaches Formal programme evaluation [18] Survey data [68, 86] Written feedback [68] Semi-structured individual interviews [105] Ungraded diary entries [99] Structured reflection report [116] Questionnaire [101, 116] Validated wellness scales [106] Focus group interviews Observation of group interactions [118]

Structured group reflection programs were predominantly organized with planning and specific guidelines, emphasizing key elements such as the preparation process, which often time entails pre-readings, communicating objectives [68], and establishing ground rules [104, 105]. Group sizes often varied between smaller groups, which allowed intimate and in-depth discussions [62, 126, 129–131]; and larger groups which allowed for a wider range of perspectives [21, 119, 132]. Frequency of these sessions varied between once-off sessions [130] to regular meetings [18, 132]. Group reflective programs utilized different modalities such as in-person meetings [21, 61, 126, 133] and online meetings [25, 128, 134] which are scaffolded by other activities reviewing video playback [104, 106].

With regards to the assessment of group reflective programs, the most common method used were feedback surveys [18, 86, 101, 105, 109, 116, 133, 134] and questionnaires [101, 116] to gather feedback and insight into the effects of the programs. Few articles mentioned the use of assessments such as evaluation of portfolios [18] or ungraded diary entries [99], or analysing interview transcripts relating to the group reflective program [61].

### Domain 5: benefits and challenges

Group reflections have professional and personal benefits. On a personal basis, group reflections facilitate self-assessment and self-development, reduce anxiety [24, 119, 136], stress and burnout [18, 24, 81, 89, 137] and enhance compassion and empathy amongst participants [18, 119, 130, 132, 137].

At a professional level, group reflections strengthen shared mental models and a sense of community [61, 86, 100, 130, 135, 136], build ties with peers [138] and remove hierarchy [18, 24, 82]. A summary of these benefits is included in Table 8.

**Table 8:** Benefits of group reflections

**Professional**	
Supporting professional formation of physicians	Improvement of self through sharing of reflections and receiving feedback • Identify good practices and performance gaps [89, 106, 129, 132, 136–142]. • Create strategies to refine future performances through facilitated discussion [81, 89, 109, 136, 138, 143] • Promotes expertise [115] • Improve decision-making principles [109] • Promotes self-efficacy [136] • Increased job satisfaction
	Identity formation through exploration of emotions • Linking the affective state to cognitive abilities [18] • Fosters learning and helps participants reflect on both their personal and professional values and judgment [18, 89, 124, 136, 144, 145]. • Exploring strong emotional responses, be it positive or negative [61]. • Attitudes and perceptions developed through interaction with others [29].
	Provides a way of thinking and analysing practice [29, 122, 126, 129, 131, 137, 146].
	Increased competence, confidence or security in performing functions [24, 130]
	Becoming more resilient [137]
Improved interpersonal and professional skills	Improved communication skills between healthcare professionals and with patients [20, 22, 24, 82, 109, 124, 136, 147]. • Realized importance of interprofessional teamwork • Active listening • Becoming systematically able to initiate dialogues with team members, leading to a collective basis for decision-making • Helps to understand own and others behaviours and responses.
	Improved clinical reasoning and decision making [109, 129] • Reflection on clinical situations or incidents to rationalize behaviour retrospectively • Application of the reflective experiences into real-world experiences.
	Development of soft skills • Development of empathy [18, 119, 130]
	Patient-centred care [119] • Become more aware of patient autonomy and respecting individuals • Importance of trust in relationships
	Improvement in patient outcomes [147].
As a tool for learning enhancement	Sharing of reflections • Understanding other perspectives and ideas [21, 29, 135, 136] • Encourages providing honest feedback on skills and professional qualities due to a conducive environment [119].
	Deeper understanding of skills • Development of discussion skills, exploration, sharing and reflection upon experiences [28, 84, 114, 118–120, 124, 139]. • Allows learners to synthesize content and relate them to concepts [129, 132].
	As another avenue for students to engage in learning in addition to more traditional methods in classrooms • Enhanced didactic learning [114]
	Participants have gained a broader perspective, with the attitude change and seeing the value in different opinions or utilizing other’s perspectives [24, 29, 61, 82, 148].
	Participants experiences are improved and deepened [149].
Team	Felt a sense of community and connectedness [61, 86, 100, 130, 135, 136].
	Fosters meaningful relationships between participants [86]. • It builds morale among group members [24, 81, 89, 105, 124]. • Strengthens team cohesiveness [89]
	Promotes peer support networks [138]
	Strengthening shared mental models [122]
	Feeling validated as they were not alone in experiencing these reactions [21, 82, 106, 137].
	Removal of hierarchy [18, 24, 82]
	Individual participants can react and build on other’s responses [29, 150]
**Personal**	
Self-understanding	Recognition of personal growth and enhancement of professional values [86, 119, 135]. Increases self-awareness [18, 24, 60, 109, 114, 132, 137]. • Insights into strengths, weaknesses and learning needs [25, 89] • Increased awareness of own assumptions about patient care [25, 134]. • Acknowledgement of vulnerabilities in an open, safe environment [18, 61] • Questioning of personal beliefs and actions [109]
Enhanced self-assessment	Identification of strengths and weaknesses • Greater ease with receiving critical feedback from others [61, 136] • Critical reflection of oneself, encouraging both cognitive and emotional self-awareness of beliefs, values and attitudes [25, 119].
Personal impact	Participants felt appreciated [28, 110].
	Provide emotional support and helping to process responses • Decreases stress and preventing burnout [18, 24, 81, 89, 137]. • Able to express emotions rather than keeping it in while reflecting alone [137, 151]. • Decreased anxiety [24, 119, 136]
	Increased compassion and retention of empathy [18, 119, 130, 132, 137].
	Participants felt more motivated [84, 106, 130]
	Participants felt safe [18, 28, 110, 136, 152].
	Promotes hope [136]

The challenges surrounding group reflections may be broadly categorised into structural and participant considerations. Structural considerations hinge on the presence of a formal and organised approach [89, 104, 124, 136, 138, 139, 153, 154]. Poor longitudinal support [62, 89, 109, 141, 151, 155, 156], and a lack of long term appraisals of the effects of this intervention [29, 118, 150] may hinder holistic development, impacting the skills, attitudes and well-being of participants. The lack of a formal structure compromises facilitator/tutor recruitment [55, 89, 123, 149, 157] and training [104, 109, 115, 123, 124, 138, 141, 154, 156, 158], and the provision of protected time [81, 84, 86, 89, 104, 109, 118, 153, 156, 159]. The lack of a formal program and an organised approach also compromise longitudinal oversight of participants and the program [28, 67, 110, 138, 140, 141, 154, 160] making it predisposed to resource limitations [153, 161] and unconducive practice environments [84, 109, 122, 124, 162].

Participant considerations include concerns over privacy [24, 89, 109, 158, 163], anonymity [162], and vulnerability [67, 109] within a group, as well as managing team dynamics [28, 29, 61, 67, 118, 127, 135, 150, 152, 164], negative emotions [21] and criticism [82, 122, 124, 165] within such settings. These concerns are heightened in mixed groups with participants from different specialities, backgrounds and settings [26], particularly when participants are unfamiliar with one another [22, 127]. Hierarchy and deference to elders may also inhibit sharing, interactions [22, 127], and the disclosure of views that may contradict others [139].

### Iterative stage

As part of the iterative process of the SEBA methodology, members of the expert team shared their experiences with group reflections to help contextualise the data and inculcate practical considerations (Table 9).

**Table 9:** Expert experience with group reflections

Characteristics	Palliative care clinical postings which are part of the formal medical school training program.	Group reflections following the viewing of “a good death”, a recording of a local play set in a hospice.	Group reflections of medical officers following training on breaking bad news.	Interprofessional case discussions involving nurses, Social workers and physicians in a home care unit.
Population	A group of between two to 12 peers from Duke-NUS or NUS medical school students in the final years of medical school.	8–12 DUKE-NUS medical students. These clinical groups are mature and the participants have been working with one another for at least a few months.	Three to four medical officers and residents in Oncology and Palliative Care.	Five to nine professionals. Facilitated by the senior physician.
Format	Case presentation of a case discussed or seen by the students. Compulsory aspect of the posting. The students are aware of the format and the expectations surrounding the session.	Group debrief facilitated by a medical humanities expert and one clinician. The students are provided with specific questions regarding a specific scene from the play.	Compulsory aspect of the oncology posting. Protected time from clinical work provided.	Ad Hoc discussions that may follow difficult cases, complex care and deaths. These discussions occur online.
Duration	30–60 min depending on the number of peers. Facilitated by a senior clinician.	60 min	30 min following the session	30 min
Settings	Physicians lounge or designated training room.	Teaching room	Teaching room	Online
Lessons learnt	Smaller groups, well-established groups who have known and worked together for some time. Peers with similar sociocultural backgrounds worked better. Discussions on facets that were experienced by all the participants brought about deeper and greater sharing. Sharing was enhanced when the facilitator was known to the students and who was present at the episode being reflected upon.	The session often initiates personal insights and participation from the other participants. The nature of the discussion engenders a respectful sharing environment and the presence of a medical humanities expert who is not a clinician reduces the hierarchy in the sharing and interactions.	The small groups facilitate sharing. This sharing is enhanced when the peers know and have worked together for a while.	These groups of participants from different backgrounds share perspectives but remain largely within the confines of their specialist field of knowledge. Personal sharing is usually limited to the emotions surrounding the episode being discussed.
Impact	As a matter of routine, all students are asked what they had learnt, what has changed the way they think, and what might affect their practice in the future. These questions bring about deeper reflection and encourages enduring effects.	Similar to clinical discussions. These sessions also surface personal issues that invite personal debriefs and sometimes referrals for further support from the Student Affairs team.	Similar to clinical discussions	Sharing may be limited given the professional environment and the culture of the team. It may also be limited by the presence of professionals of difference experience, specialities and seniority.

In our experience with group reflections, participants describe, discuss, and enrich a common or shared experience with personal, professional, practical, team, sociocultural and administrative insights, and perspectives. In many instances, the facilitator plays an active part in this process, acting as a source of clinical, professional, ethical, legal, and organisational knowledge that may be used to anchor the discussion. The facilitator also plays a key role in focusing the discussion, engaging all the participants and ensuring that the reflective process occurs within a safe environment that is conducive to the sharing of personal, private and emotional information [19, 20, 26, 28–30, 99, 117, 120]. A safe environment is one where participants see “one another as equal relational partners", and "question assumptions, power dynamics and structural inequities beyond medicine” [23–25].

## Discussion

In addressing its primary research question on “*what is known about group reflections in medical education?*”, this SSR in SEBA reveals a growing role for group reflections in medical education, driven by growing reports of unprofessional conduct [166], poor communications [167] and inadequate mentoring support in medicine. This trend is exacerbated by a shortage of trained facilitators to support reflections, which has been further amplified by the challenges posed by the pandemic. Flourishing in its nurturing of PIF [168], interpersonal and professional skills, group reflections provide timely, personalised senior and peer support, integrates different perspectives and fosters cohesive working environments in medicine and the allied health specialities [31, 38, 169–171]. Yet the data suggests that the practice and effects of it vary, which is underlined by the presence of different forms of group reflections focused on varying depths of reflections guided by a mix of current theories of reflections.

Incorporating data from the review with practical experiences of group reflections (Table 9) demonstrate that group reflections can be shown to pivot on individual, group and environmental considerations.

### Individual considerations

**Fig. 3:**
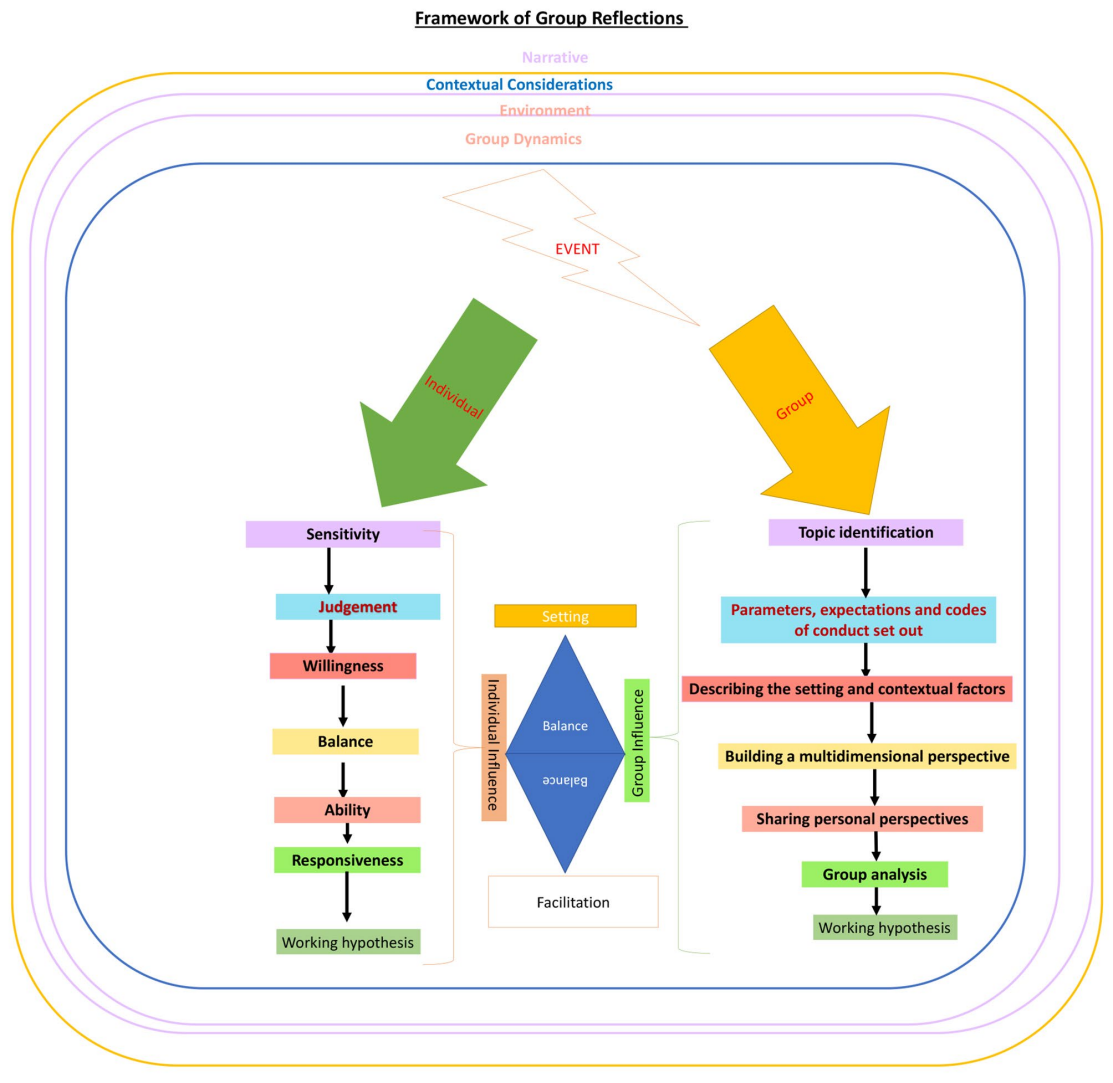
Framework of group reflections

 Given the scarcity of information on the individual aspects of group reflections within the current data, Krishna’s model of Reflective Writing (KmRW) from Lim et al. [5]’s review on the subject was adopted (Fig. 3). The KmRW was based on the same guiding theories and practice used in group reflective practice and provides evidence-based perspectives of the individual’s experiences with reflections, focusing on the clinician’s role in the reflective process, beginning with the clinician’s sensitivity to the presence of experiences and/or threats to belief systems [5, 172]. Rooted within the clinician’s self-concept of personhood, the belief system is shaped by and manifested in the sense of identity and in their feelings, attitudes, thoughts, decision making, and conduct. To preserve the current sense of self-identity, the clinician seeks to confront these threats to their self-concepts of personhood. This raises the notion as to their willingness to address these issues, their ability to judge and balance ramifications as a result of actions, omissions and partial actions that may arise within their personal, psychosocial, clinical, professional, research, academic, administrative, and situational context and their capability to adapt their belief system in response to the insights gained. The clinician’s *‘responsiveness’* highlights the individual’s capacity to attend and adapt their practice in light of the insights gained. The elements of the individual aspect of the reflective process are featured on the left aspect of Fig. 3.

### Group and environmental considerations

Our data coupled with expert experiences with group reflections (Table 8), spotlights the influence of group dynamics and the structure of the reflective process and its environment, setting and contextual factors. These facets shift attention from individual ‘*judgement’, ‘willingness’, ‘balance’, ‘ability’ and ‘responsiveness’* to group-determined areas that include.

- the topic for discussion (this includes what, why and how the topic for reflection was identified),
- participation (this includes setting a basic level for participation, an expectation on conduct and interactions that influences group dynamics, and the sense of ‘safety’ the individual feels about sharing).
- willingness to reflect and share their reflections (aside from levels of participation, and establishing a safe environment for sharing, the group reflective process must motivate individual sharing and imbue the discussion with their narratives, experiences, and emotions).
- willingness and ability to analyze the experience.
- creating a ‘*working hypothesis’*.

Acknowledgement of these group, practice and structural considerations suggest a wider range of factors impacting group reflections than what is encompassed by the KmRW. Group reflections that confine discussions to a specific area of interest; establish parameters on the nature of interactions; knit together the various perspectives; and synthesize a cogent narrative of events replete with contextual, emotional, sociocultural and practical factors; underscore how organizing group reflective processes influences both experiences and results of the reflection. These features are delineated on the right side of Fig. 3.

These considerations draw attention to the secondary research question “*How are group reflections structured, assessed and supported in medical education*?”. Accrued data and expert opinions suggest that group reflections must build upon a consistent approach; agreed upon codes of practices, levels of participation, roles, and responsibilities; aligned expectations; effective facilitation and a nurturing environment [104, 105, 119]. The invitation to participate emphasizes privacy and includes information on the number of participants, the facilitator’s backgrounds, the setting, the duration of session, and how information will be shared [24, 89, 109, 158, 163]. The participants are also given access to personal debriefs, counselling and/or psychological support after the session [30, 67, 121, 133, 136, 138, 150, 152, 154, 157, 173, 174]. Groups should ideally comprise of participants with similar levels of experience or seniority, or individuals who are comfortable with sharing and discuss their views, experiences, insights and lessons learnt. The program should be facilitated by a trained and impartial facilitator who may be an expert in the field that can manage group dynamics, guide the synthesis of a cogent narrative, offer insights and personalised support should the need arise and debrief the participants individually if needed [21, 24, 28, 30, 89, 109, 121, 156, 162, 163, 175]. The session should be carried out in a ‘safe’ and appropriate setting that will be conducive to open sharing [29, 86, 109, 121–123, 134, 136, 143, 150, 154, 156, 176]. The session should be ring-fenced or be part of the ‘protected time’ for reflections during the training program [28, 30, 121–123, 137].

The assessment of group reflection programs is critical to understanding their impact on learners and evaluating their effectiveness. These assessment methods provide valuable feedback to educators and the continuous improvement of group reflection programs. The most common method of evaluation used in the included studies were feedback surveys and questionnaires which are valuable in gauging participant satisfaction and identifying the strengths and weaknesses of the program design. Other methods such as the written reflections, interviews and wellness scales were used in addition to these feedback methods to further explore participants’ experiences and insights that were gained through the reflective process. A future endeavour could be towards the development of a portfolio for medical learners to acknowledge the impact of these reflections on the well-being of participants, providing an avenue for feedback and improvement.

### Limitations

Despite evaluation of the search process by the expert team, including only English language articles and excluding grey literature, the risk of failing to capture important articles is present. Concurrently, scrutinising publications in English skews the attention onto Western practice where distinct sociocultural, practice, education and healthcare considerations may limit the applicability of these findings in settings beyond the North American and European setting.

The purposeful selection of search terms and the employment of a wide range of databases broadened the approach to obtaining essential publications. However, the inclusion of a wide range of search terms and articles and the exclusion of non-healthcare settings may limit our analysis of the conceptualisation of the phenomenon.

Although thematic analysis was conducted by independent members of the team to improve the credibility and reliability of the data, inherent bias cannot be eliminated entirely. Perhaps most significantly is the conflation of terms and practices surrounding group reflections and debriefs.

## Conclusions

Group reflections emphasize the need for targeted discussions, clear guidelines, and the incorporation of various perspectives to synthesize a comprehensive understanding of medical education. However, this review highlights the challenges in ensuring longitudinal support and appraisals, which are crucial in sustaining professional development. Aside from the need for further research into faculty training and structuring a consistent approach, future development of group reflections should focus on establishing robust frameworks for assessment, fostering ongoing support structures and integrating technological advancements to enhance the efficacy of reflective processes. A comprehensive approach considering both immediate and long-term impacts of group reflections is essential to cultivating well-rounded and empathetic healthcare professionals.

## Data Availability

All data generated or analysed during this review are included in this published article.
